# Manganese(II) Complexes with Schiff Bases Immobilized on Nanosilica as Catalysts of the Reaction of Ozone Decomposition

**DOI:** 10.1186/s11671-015-1179-6

**Published:** 2015-12-08

**Authors:** Tetyana Rakytska, Alla Truba, Evgen Radchenko, Alexander Golub

**Affiliations:** Odesa I.I. Mechnikov National University, 2 Dvoryanska str., Odesa, 65082 Ukraine; Taras Shevchenko National University of Kyiv, 64 Volodymyrska str., Kyiv, 01601 Ukraine; National University of Kyiv-Mohyla Academy, 2 Skovorody str., Kyiv, 04655 Ukraine

**Keywords:** Nanosilica, Manganese(II) complexes, Schiff bases, Catalytic ozone decomposition

## Abstract

In this article, we submit the description of synthesis and identification of manganese(II) complexes with pyrogenic nanosilica-immobilized (*d*_av_ = 10 nm; *S*_sp_ = 290 m^2^/g) hydroxyaldimine ligands $\left(\mathrm{M}\mathrm{n}{\left(\mathrm{L}\right)}_2/\overline{\mathrm{Si}}\right)$: salicilaldiminopropyl (L1); 5-bromosalicilaldiminopropyl (L2); 2-hydroxynaphtaldiminopropyl (L3); 2-hydroxy-3-methoxybenzaldiminopropyl (L4); 2-hydroxy-3,5-dichloroacetophenoniminopropyl (L5); and 4-hydroxy-3-methoxybenzaldiminopropyl (L6). The ligands and complexes were characterized by UV-VIS and IR spectrometry. Nanocomposites consisting of complexes $\mathrm{M}\mathrm{n}{\left(\mathrm{L}\right)}_2/\overline{\mathrm{Si}}$ showed a high catalytic activity in low-temperature ozone decomposition in the range of concentrations between 2.1 × 10^−6^ and 8.4 × 10^−6^ mol/l. The number of catalytic cycles increased for isostructural pseudotetrahedral complexes $\mathrm{M}\mathrm{n}{\left(\mathrm{L}\right)}_2/\overline{\mathrm{Si}}$ (L1–L5) in the following order: Mn(L3)_2_ >> Mn(L4)_2_ > Mn(L1)_2_ > Mn(L2)_2_ > Mn(L5)_2_. In the case of pseudooctahedral complexes with L6, the change of coordination polyhedral does not influence the kinetics and stoichiometric parameters of the reaction.

## Background

Dissolved complexes of 3d metals with Schiff bases, especially Mn(II, III) and Co(II) complexes, are used successfully as catalysts of many oxidation reactions of organic compounds [1]. Manganese-Schiff base complexes like square planar Mn(salen) (where salen is *N,N*′-bis(salicilald)ethylenediiminato(2-)anion) have shown considerable promise in superoxide dismutase (SOD) and catalase-like activity which could be a perspective for the creation of new medicines with wide applications [2]. However, difficulties of complex dimerization and extraction of the product and catalyst from solution, as well as the endeavor of modeling natural enzymatic systems, stimulated the research of synthesis of immobilized homogeneous catalysts of oxidation including complexes with Schiff bases [3]. At the beginning, synthetic polymeric materials based on styrene were widely used as carriers of the complexes [4–8]. There are efforts to use, instead of synthetic polymers, natural polymers as carriers for Schiff base complexes with, for example, chitosan that is easily degraded and well combined with human blood, which makes them promising in biomedical practice [9, 10]. Although anchored complexes demonstrated positive properties compared with homogeneous analogs (the raise in catalytic activity and the number of catalytic cycles, selectivity) [6, 7], however, they had lower stability after increasing the temperature of the reaction. Due to this, other available carriers characterized by chemical, thermal, and mechanical stability can be widely applied. As such carriers, different forms of activated carbon [11–14], dispersed silica of various origins [15–18], zeolites [19–23], and ordered mesoporous molecular sieves, for example, МСМ-41 and МСМ-48 [24], can be used.

These carriers are characterized by a high internal surface; therefore, immobilization of complexes takes place on both the outer and inner surfaces. In the latter case, an access of reagents to the immobilized complexes and, consequently, the kinetics of the reaction are determined by internal diffusion factors. Intradiffusious inhibition of the reaction with metal-Schiff base complexes can be avoided if non-porous pyrogenic nanosilica (aerosil) with developed outer surface is used as a carrier [25]. It should be noted that manganese(II) becomes manganese(III, IV) in the course of the synthesis of immobilized complexes with Schiff bases on the noted carriers, except nanosilica [25] and aminated silica [17].

Analysis of data obtained as a result of the study of catalytic properties of 3d metal complexes with Schiff bases in the oxidation of organic compounds [15, 17, 18, 20–22, 24, 26–28] and decomposition of ozone [29, 30] leads to the conclusion that the catalytic activity of the immobilized metal complexes in redox reactions can be controlled by the following: (i) optimization of the structural characteristics of the carrier and the method of synthesis for obtaining the homogeneous structure and composition of immobilized complexes, (ii) changes in the geometric configuration of an immobilized complex, and (iii) redistribution of the electron density at the central atom and the ligand leading to a significant change in redox potential of M^n+1^/M^n+^ pair and, hence, to the reactivity and catalytic activity of the complexes.

All the abovementioned aspects of the synthesis and catalytic activity of nanosilica-immobilized manganese(II)-Schiff base complexes in the reaction of ozone decomposition have not been studied.

The aim of this work is to study the influence of the nature of pyrogenic nanosilica-immobilized Schiff bases on the structure and catalytic activity of manganese(II) complexes in the reaction of ozone decomposition.

## Methods

Pyrogenic nanosilica (model А-300, *d*_av_ = 10 nm, *S*_sp_ = 290 m^2^/g) was purchased from VAT Oriana (Kalush, Ukraine) and was used for synthesis of γ-aminopropylsilica (APS) (the concentration of aminopropyl groups, [H_2_NC_3_H_6_−], is 0.7 mmol/g SiO_2_) by routine procedure [31, 32] used for the synthesis of ligands. Immobilized Schiff bases (L1–L6) salicilaldiminopropyl (L1); 5-bromosalicilaldiminopropyl (L2); 2-hydroxynaphtaldiminopropyl (L3); 2-hydroxy-3-methoxybenzaldiminopropyl (L4); 2-hydroxy-3,5-dichloroacetophenoniminopropyl (L5); and 4-hydroxy-3-methoxybenzaldiminopropyl (L6) were obtained from APS by known methods by the following scheme [31].
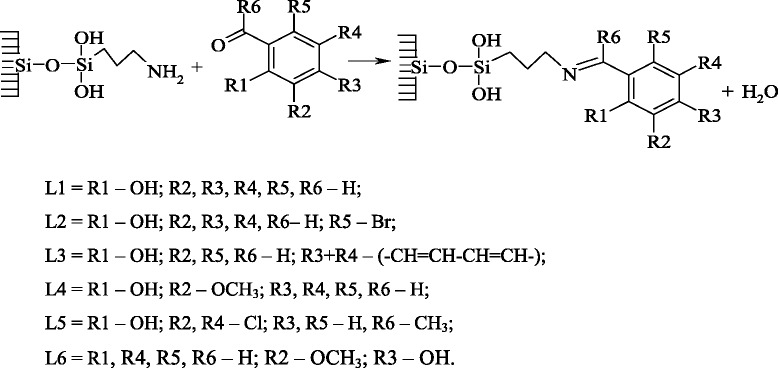


Complexes $\mathrm{M}\mathrm{n}{\left(\mathrm{L}\right)}_2/\overline{\mathrm{Si}}$ (L = L1–L6) were obtained by sorption on modified nanosilica from the absolute alcohol solution of dehydrated MnCl_2_; the concentration before sorption was 10^−2^ mol/l, and Мn^2+^(solution):L(surface) ratio was 1:2. Concentrations of the immobilized ligands and chemical compositions of the obtained complexes are shown in Table 1.

**Table 1:** Complexes of Mn(II) with Schiff bases immobilized on nanosilica

Sample	C_L_ × 10^4^, mol/g	Concentration of Mn(II)	
		C_Mn2+_ × 10^4^, mol/g	C_Mn2+_, wt%
Mn(L1)_2_	7.0	0.60	0.30
Mn(L2)_2_	7.0	0.80	0.44
Mn(L3)_2_	7.0	0.17	0.09
Mn(L4)_2_	7.0	0.80	0.46
Mn(L5)_2_	5.0	1.60	0.66
Mn(L6)_2_	7.0	0.70	0.38

IR spectra were recorded by a Fourier transform Perkin Elmer Spectrum BX FT-IR instrument (400–4000 cm^−1^) from transparent tablets obtained by the pressing of bare samples and the addition of KBr as well.

Diffuse reflectance spectra were recorded at room temperature in the wave number range of 30,000–11,000 cm^−1^ by a Specord M-40 in stainless steel cells where the samples were pressed in.

The catalyst samples (*m* = 0.2 g) were tested using a gas flow setup with a fixed-bed reactor at 20 °C, relative humidity of 65 %, and ozone-air mixture (OAM) linear velocity of 6.2 cm/s. Ozone decomposition was monitored by measuring the final ozone concentration $\left({C}_{{\mathrm{O}}_3}^{\mathrm{f}}\right)$. The initial ozone concentrations $\left({C}_{{\mathrm{O}}_3}^{\mathrm{in}}\right)$ and ${C}_{{\mathrm{O}}_3}^{\mathrm{f}}$ were measured either by a Tsyclon-Reverse optical gas analyzer (detection limit of 1 mg/m^3^).

The reaction rate (*W*) was calculated based on the data of ozone concentration change after passing the OAM through the static bed of the catalyst using the following equation:

1 $$ W=\frac{\omega \left({C}_{{\mathrm{O}}_3}^{\mathrm{in}}-{C}_{{\mathrm{O}}_3}^{\mathrm{f}}\right)}{m_{\mathrm{cat}}},\mathrm{mol}/\left(\mathrm{g}\cdotp \mathrm{s}\right), $$

where *ω* = 1.67 × 10^−2^ is the OAM volume flow rate, l/s; ${C}_{O_3}^{in}$ and ${C}_{O_3}^f$ are the initial and final ozone concentrations, respectively, in the OAM, mol/l; and *m*_cat_ is the weight of a catalyst sample, g.

The initial reaction rate, *W*_in_, was defined as *W* after 1 min of experiment.

The kinetic constants (*k*_1_) at the beginning of the experiment (after 5–10 min) and at 50 % conversion of ozone (*k*_1/2_) were found from the first-order rate equations:

2 $$ {k}_1=\frac{1}{\tau } \ln \frac{C_{{\mathrm{O}}_3}^{\mathrm{in}}}{C_{{\mathrm{O}}_3}^{\mathrm{f}}},{\mathrm{s}}^{-1}, $$

3 $$ {k}_{1/2}=\frac{0.69}{\tau_{1/2}},\kern0.5em {\mathrm{s}}^{-1}. $$

where *τ*_1/2_ is the ozone half-conversion time.

The amount of ozone that entered the reaction over the course of the experiment (*Q*_exp_, moles of О_3_) was calculated as the area under the corresponding ozonogram plotted as a $\varDelta {C}_{{\mathrm{O}}_3}$ vs. *τ* function. This magnitude was used for the calculation of the following stoichiometric coefficients: *n*_L_ = *Q*_exp_/*Q*_L_ characterizing the number of moles of ozone per mole of an immobilized ligand (*Q*_L_); *n*_Mn_ = *Q*_exp_/*Q*_Мn_ giving the number of moles of ozone per mole of manganese(II) in the complex; and *n*_СН_ = *Q*_exp_/*Q*_СН_ showing the extent of mineralization of the hydrocarbon part of a ligand (*Q*_СН_ is the number of moles of ozone required for complete oxidation of the hydrocarbon part of a molecule calculated relying on the stoichiometry of reactions (4)–(8)).

4 $$ \mathrm{L}1/\overline{\mathrm{Si}}\kern1em 3{\mathrm{C}}_7{\mathrm{H}}_6+17{\mathrm{O}}_3 = 21\mathrm{C}{\mathrm{O}}_2 + 9{\mathrm{H}}_2\mathrm{O} $$

5 $$ \mathrm{L}2/\overline{\mathrm{Si}}\kern1em 6{\mathrm{C}}_7{\mathrm{H}}_5+33{\mathrm{O}}_3 = 42\mathrm{C}{\mathrm{O}}_2+15{\mathrm{H}}_2\mathrm{O} $$

6 $$ \mathrm{L}3/\overline{\mathrm{Si}}\kern1em 3{\mathrm{C}}_{11}{\mathrm{H}}_8 + 26{\mathrm{O}}_3 = 33\mathrm{C}{\mathrm{O}}_2 + 12{\mathrm{H}}_2\mathrm{O} $$

7 $$ \mathrm{L}4/\overline{\mathrm{Si}}\kern0.5em \mathrm{and}\kern0.5em \mathrm{L}6/\overline{\mathrm{Si}}\kern1em 3{\mathrm{C}}_8{\mathrm{H}}_8 + 20{\mathrm{O}}_3 = 24\mathrm{C}{\mathrm{O}}_2 + 12{\mathrm{H}}_2\mathrm{O} $$

8 $$ \mathrm{L}5/\overline{\mathrm{Si}}\kern1em 3{\mathrm{C}}_8{\mathrm{H}}_6+19{\mathrm{O}}_3 = 24\mathrm{C}{\mathrm{O}}_2+9{\mathrm{H}}_2\mathrm{O}. $$

## Results

### Composition and Structure of Mn(II) Complexes

The spectral characteristics of Schiff bases immobilized on nanosilica, $\mathrm{L}/\overline{\mathrm{Si}}$ (L = L1–L6), and their complexes with manganese(II), $\mathrm{M}\mathrm{n}{\left(\mathrm{L}\right)}_2/\overline{\mathrm{Si}}$, are summarized in Table 2. In the IR spectra of all complexes except $\mathrm{M}\mathrm{n}{\left(\mathrm{L}5\right)}_2/\overline{\mathrm{Si}}$ was observed a low-frequency shift of a peak (5–15 cm^−1^) characterizing stretching vibrations of the imino group (С=N) compared with free ligands that indicates an electron density transfer in Mn–N=C bond. Furthermore, in the $\mathrm{M}\mathrm{n}{\left(\mathrm{L}4\right)}_2/\overline{\mathrm{Si}}$ complex, alongside with a low-frequency shift of a *ν*_С=N_ band at 1650 cm^−1^, a peak with lower energy appears at 1620 cm^−1^ which can indicate the finding of ligand L4 in two different configurations. In the case of ligand L6 characterized by two absorption peaks at 1650 and 1600 cm^−1^, a complex formation results in the high-frequency shift of the first of them whereas the second one remains without alteration.

**Table 2:** Spectral characteristics of nanosilica-immobilized Schiff bases and $\mathrm{M}\mathrm{n}{\left(\mathrm{L}\right)}_2/\overline{\mathrm{Si}}$ complexes

Ligand complex	Wave numbers of peak maximums, cm^−1^	
	*ν*_C=N_	*ν*_π − π_*
L1	1635	24,600
$\mathrm{M}\mathrm{n}{\left(\mathrm{L}1\right)}_2/\overline{\mathrm{Si}}$	1630	24,900
L2	1642	29,700; 23,900; 19,400
$\mathrm{M}\mathrm{n}{\left(\mathrm{L}2\right)}_2/\overline{\mathrm{Si}}$	1630	29,800; 24,000; 19,600
L3	1640	23,600; 25,400
$\mathrm{M}\mathrm{n}{\left(\mathrm{L}3\right)}_2/\overline{\mathrm{Si}}$	1630	23,600; 25,400
L4	1650	23,600
$\mathrm{M}\mathrm{n}{\left(\mathrm{L}4\right)}_2/\overline{\mathrm{Si}}$	1645; 1620	24,800; 25,000
L5	1650	23,960
$\mathrm{M}\mathrm{n}{\left(\mathrm{L}5\right)}_2/\overline{\mathrm{Si}}$	1630	24,300
L6	1650; 1600	25,000
$\mathrm{M}\mathrm{n}{\left(\mathrm{L}6\right)}_2/\overline{\mathrm{Si}}$	1665; 1600	27,200; 25,600

In UV-VIS spectra after complexation, the high-frequency shift occurs for the peaks characterizing the electron density transfer in ligand *ν*_*π* − π*_, compared with a free immobilized Schiff base. Furthermore, in some cases, additional bands appear. Similar changes were observed in the spectra of complexes $\mathrm{C}\mathrm{o}{\left(\mathrm{L}\right)}_2/\overline{\mathrm{Si}}$ and $\mathrm{C}\mathrm{u}{\left(\mathrm{L}\right)}_2/\overline{\mathrm{Si}}$ (L = L1–L6) [29, 30].

Sometimes, oxidation of manganese(II) to manganese(III) was observed, which was evidenced by the appearance of an asymmetrical low-intensity shoulder in the visible part of spectrum at 18,500 cm^−1^ (510 nm) that can be relied to a d-d electron transfer in complexes of the square-pyramidal geometry [33–35].

**Scheme 1:**
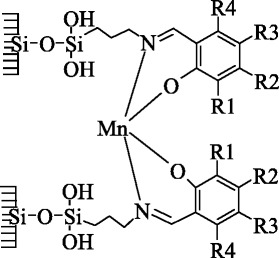
Structure of the manganese(II) complexes with ligands L1-L5

The results of our research have shown that in the visible part of spectrum for all synthesized Mn(II) complexes, a d-d electron transfer is not detected which can be one of the proofs of a stable oxidation state of the complexing ion. A similar example is the synthesis of Mn(II)(salen) complexes on porous amino silica gel (Si-NH_2_) [17]. A comparison of the spectral data for similar Cu(II) and Co(II) [29, 30] complexes and $\mathrm{M}\mathrm{n}{\left(\mathrm{L}\right)}_2/\overline{\mathrm{Si}}$ complexes permits to conclude that, for manganese(II), polyhedron N_2_O_2_ also realizes in the ligand field of L1–L5 (Scheme 1).

Compared with a square planar structure for individual crystalline Schiff base complexes, complexes immobilized on a silica surface suffer a tetrahedral distortion due to the anchoring of a ligand foot to the nanoparticle surface, and that can be one of the causes of a raise in their reaction activity. The tetrahedral distortion of immobilized bis-ligand complexes (except the pseudo octahedral complex with L6) is confirmed by modeling the surface clusters by molecular-mechanical methods (MM2) and by semi-empirical quantum chemistry methods (PM3) as well.

**Scheme 2:**
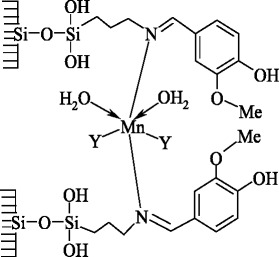
Structure of the manganese(II) complex with ligands L6

L6, unlike the isomeric L4, cannot form chelate cycles, and coordination with the Mn(II) ion is carried out only by an azomethine group (Scheme 2), which leads to the high-frequency shift of the peak assigned to C=N group vibrations and the ligand charge transfer (*π* − π*).

Thus, all synthesized $\mathrm{M}\mathrm{n}{\left(\mathrm{L}\right)}_2/\overline{\mathrm{Si}}$ complexes differ by both the nature of the Schiff base immobilized on nanosilica and the structure of a coordination polyhedron. It is of interest to test the influence of the distinctive factors on the activity of manganese(II) complexes in the reaction of ozone decomposition.

### Testing $\mathrm{L}/\overline{\mathrm{Si}}$ Nanocompositions in the Reaction of Ozone Decomposition

**Fig. 1:**
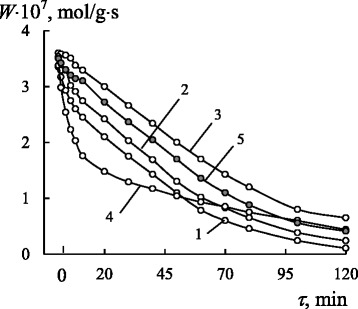
The time dependence of *W* for ozone decomposition by $\mathrm{L}/\overline{\mathrm{Si}}$ ligands: L1 (*1*); L3 (*2*); L4 (*3*); L5 (*4*); L6 (*5*) $\left({C}_{{\mathrm{O}}_3}^{\mathrm{in}} = 4.2\times {10}^{-6}\mathrm{mol}/\mathrm{l}\right)$

Ozone can interact practically with all organic compounds at low temperature. Hence, at the beginning, the kinetics of ozone decomposition by nanosilica-immobilized Schiff bases, $\mathrm{L}/\overline{\mathrm{Si}}$ (L = L1–L6), would be studied. Time (*τ*) dependences of the reaction rate (*W*) for ozone decomposition by the nanocompositions under study (Fig. 1) show that, regardless of the ligand nature, the type of kinetic curves is the same, i.e., the reaction rate decreases in time. In the case of L5 (curve 4), the reaction rate for the first 20 min decreases more drastically. The results of the kinetic and stoichiometric analysis of the obtained data will be discussed hereafter.

### Testing $\mathrm{M}\mathrm{n}{\left(\mathrm{L}2\right)}_2/\overline{\mathrm{Si}}$ Nanocompositions in the Reaction of Ozone Decomposition

**Fig. 2:**
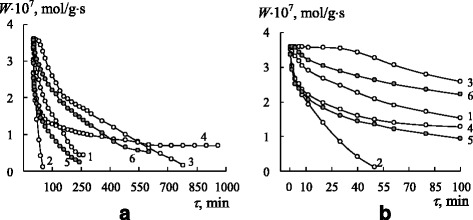
The time dependences of *W* for ozone decomposition by $\mathrm{M}\mathrm{n}{\left(\mathrm{L}\right)}_2/\overline{\mathrm{Si}}$ complexes (L = L1–L6): Mn(L1)_2_ (*1*); Mn(L2)_2_ (*2*); Mn(L3)_2_ (*3*); Mn(L4)_2_ (*4*); Mn(L5)_2_ (*5*); Mn(L6)_2_ (*6*) **a** for 1000 min and **b** for first 100 min $\left({C}_{{\mathrm{O}}_3}^{\mathrm{in}} = 4.2\times {10}^{-6}\mathrm{mol}/\mathrm{l}\right)$

Time dependences of the reaction rate in the case of ozone decomposition by $\mathrm{M}\mathrm{n}{\left(\mathrm{L}2\right)}_2/\overline{\mathrm{Si}}$ complexes (L = L1–L6) are presented in Fig. 2. It can be seen that the duration of runs varies from 50 min for the L1 ligand up to 1000 min for the L4 ligand (that run was interrupted before the final reaction rate drop). A portion of the kinetic curves for the first 100 min (Fig. 2b) allows to make the differences between the activities of the manganese(II) complexes more clear. The data of Fig. 2b show that the $\mathrm{M}\mathrm{n}{\left(\mathrm{L}2\right)}_2/\overline{\mathrm{Si}}$ complex demonstrates the lowest activity in the reaction: the reaction rate decreases to 0 $\left({C}_{{\mathrm{O}}_3}^{\mathrm{f}}={C}_{{\mathrm{O}}_3}^{\mathrm{in}}\right)$ in 50 min. A high activity is demonstrated by the $\mathrm{M}\mathrm{n}{\left(\mathrm{L}3\right)}_2/\overline{\mathrm{Si}}$ complex: the reaction rate decreases from 3.6 × 10^−7^ to 2.5 × 10^−7^ mol/(g·s) in 100 min; in other words, the ozone concentration at the reactor outlet decreases only by 50 mg/m^3^.

Manganese(II) complexes with L1, L4, L5, and L6 ligands occupy an intermediate position. A trend of ozone decomposition by manganese(II) complexes to occur in a steady-state mode can be seen in Fig. 2a. It is especially apparent for complexes containing L4 and L6 ligands. The duration of the stationary portion for $\mathrm{M}\mathrm{n}{\left(\mathrm{L}4\right)}_2/\overline{\mathrm{Si}}$ exceeds 600 min. During the reaction with ozone, the Mn(II) complexes, except for inactive $\mathrm{M}\mathrm{n}{\left(\mathrm{L}2\right)}_2/\overline{\mathrm{Si}}$, became brown or brownish black (Table 1). Such a color change indicates that Mn(II) turns into Mn(IV) in its oxide form which is a secondary catalyst of ozone decomposition less active than Mn(II) complexes: the reaction rate of ozone decomposition in the steady-state mode is low.

**Fig. 3:**
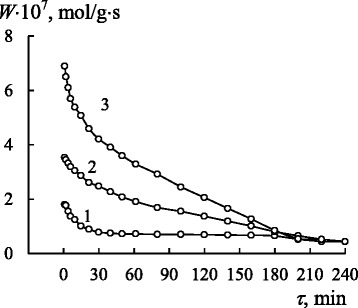
The time dependences of *W* for ozone decomposition by $\mathrm{M}\mathrm{n}{\left(\mathrm{L}1\right)}_2/\overline{\mathrm{Si}}$ at ${C}_{{\mathrm{O}}_3}^{\mathrm{in}}$ × 10^6^, mol/l: 2.1 (*1*); 4.2 (*2*); 8.4 (*3*) (C_Mn(II)_ = 6.0 × 10^−5^ mol/g)

As an example, Fig. 3 demonstrates how the reaction rate for ozone decomposition by $\mathrm{M}\mathrm{n}{\left(\mathrm{L}1\right)}_2/\overline{\mathrm{Si}}$ changes with increasing the initial ozone concentration in OAM from 2.1 × 10^−6^ to 8.4 × 10^−6^ mol/l (100–400 mg/m^3^). It is obvious that the initial reaction rate (*W*_in_) measured after 1 min of OAM passing increases in proportion to ${C}_{{\mathrm{O}}_3}^{\mathrm{in}}$. It is evidence of the first-order reaction with respect to ozone.

### Kinetic and Stoichiometric Parameters of the Reaction of Ozone Decomposition by $\mathrm{L}/\overline{\mathrm{Si}}$ and $\mathrm{M}\mathrm{n}{\left(\mathrm{L}\right)}_2/\overline{\mathrm{Si}}$ Nanocompositions

To analyze the data obtained, kinetic (*W*, *k*_1_, *τ*_1/2_, *k*_1/2_) and stoichiometric (*Q*_exp_, *Q*_СН_, *n*_L_, *n*_СН_, *n*_Mn_) parameters of the reaction were considered. Their values in the case of ${C}_{{\mathrm{O}}_3}^{\mathrm{in}}$ varying in the presence of $\mathrm{M}\mathrm{n}{\left(\mathrm{L}1\right)}_2/\overline{\mathrm{Si}}$ are summarized in Table 3. As can be seen, all stoichiometric parameters in the series increase with the rise in ${C}_{{\mathrm{O}}_3}^{\mathrm{in}}$. The first-order reaction rate constant, *k*_1_, is invariant only at the beginning of the reaction. Then, it decreases, varies within the series, and is not equal to the reaction rate constant, *k*_1/2_, corresponding to the time of half-conversion of ozone, *τ*_1/2_. The half-conversion time is shortened with ${C}_{{\mathrm{O}}_3}^{\mathrm{in}}$ increasing. These results allow the assumption that the reaction of ozone decomposition proceeds by the radical chain mechanism. Similar regularities were obtained for other $\mathrm{M}\mathrm{n}{\left(\mathrm{L}\right)}_2/\overline{\mathrm{Si}}$ complexes (L = L2–L6).

**Table 3:** Effect of ${C}_{{\mathrm{O}}_3}^{\mathrm{in}}$ on kinetic and stoichiometric parameters of the reaction of ozone decomposition by $\mathrm{M}\mathrm{n}{\left(\mathrm{L}1\right)}_2/\overline{\mathrm{Si}}$ (C_Mn(II)_ = 0.6 × 10^−4^; C_L_ = 7.0 × 10^−4^ mol/g)

${C}_{{\mathrm{O}}_3}^{\mathrm{in}}\times 1{0}^6,\mathrm{mol}/\mathrm{l}$	*W*_in_ × 10^7^, mol/(g·s)	*k*_1_ × 10^3^, s^−1^	*τ*_1/2_, s	*k*_1/2_ × 10^4^, s^−1^	*Q*_exp_ × 10^5^, О_3_ moles	*Q*_СН_ × 10^5^, О_3_ moles	*n*_L_	*n*_СН_, %	*n*_Mn_
2.1	1.7	3.6	6600	1.0	30.2	74.7	2.2	40.0	25.2
4.2	3.5	3.8	4800	1.4	42.1	74.7	3.0	56.0	38.3
8.4	7.0	3.8	3900	1.8	49.7	74.7	3.6	66.5	41.4

Table 4 summarizes the results of the kinetic study of ozone decomposition by immobilization on nanosilica Schiff bases (Fig. 1) and their Mn(II) complexes (Fig. 2) at ${C}_{{\mathrm{O}}_3}^{\mathrm{in}} = 4.2\times {10}^{-6}\mathrm{mol}/\mathrm{l}$. It can be seen that the immobilized Schiff bases can independently decompose ozone. The values of their kinetic constant *k*_1_ calculated for the first 10–20 min of the runs depend on the ligand nature and change in the order L5 < L2 < L3 < L1 < L6 < L4. To the moment of ozone half-conversion, *k*_1_ ≠ *k*_1/2_. The stoichiometric coefficient, *n*_L_, is less than 1 for L1–L3 ligands and exceeds 1 for L4–L6 ligands. Coefficient *n*_СН_ characterizing completeness of oxidation of the hydrocarbon part of the ligands does not exceed 30 %, i.e., the complete decomposition of the ligands does not occur in one cycle.

**Table 4:** Kinetic and stoichiometric parameters of the reaction of ozone decomposition by $\mathrm{M}\mathrm{n}{\left(\mathrm{L}\right)}_2/\overline{\mathrm{Si}}$ complexes $\left({C}_{{\widehat{\mathrm{O}}}_3}^{\mathrm{in}}=4,2\times {10}^{-6}\mathrm{mol}/\mathrm{l}\right)$

Sample	*k*_1_ × 10^3^, s^−1^	*τ*_1/2_, s	*k*_1/2_ × 10^4^, s^−1^	*Q*_exp_ × 10^5^, О_3_ moles	*Q*_CH_ × 10^5^, О_3_ moles	*n*_L_	*n*_CH_, %	*n*_Mn_
L1	3.8	1800	3.8	11.1	74.7	0.8	15.0	–
Mn(L1)	3.8	4800	1.4	42.1	74.7	3.0	56.0	38.3
L2	2.8	960	7.2	10.6	72.3	0.8	15.0	–
Mn(L2)	2.4	600	11.5	6.4	72.3	0.5	9.0	4.0
L3	3.2	1600	4.3	12.3	116.7	0.9	10.5	–
Mn(L3)	–	13,800	0.5	127.2	116.7	9.1	109.0	374.1
L4	6.2	3300	2.2	26.0	86.4	1.8	30.0	–
Mn(L4)	6.0	1680	4.1	101.0	86.4	7.0	117.0	63.1
L5	1.3	240	28.8	13.2	45.0	1.8	29.0	–
Mn(L5)	1.6	1080	6.4	35.3	45.0	4.7	78.0	15.0
L6	5.6	2820	2.4	19.6	84.0	1.4	23.0	–
Mn(L6)	5.1	10,200	0.7	93.7	84.0	6.7	111.0	66.8

The values of *k*_1_ are similar for the free ligands and for their complexes with manganese(II). It can be caused by the fact that, at the beginning of the reaction, an ozone molecule interacts with the same reaction site, i.e., a C=N group. *k*_1/2_ values do not coincide with *k*_1_ ones indicating a change in the reaction order with respect to ozone due to occurring side chain-radical reactions.

Stoichiometric coefficients *n*_L_ increase for the manganese(II) complexes as compared with the corresponding ligands; the fact that *n*_Mn_ >> 1 (the maximum value is observed for $\mathrm{M}\mathrm{n}{\left(\mathrm{L}3\right)}_2/\overline{\mathrm{Si}}$) suggests a multiple participation of Mn(II) in the reaction of ozone decomposition and a catalytic behavior of the complexes.

A catalytic effect of manganese appears also in the increase of *n*_CH_ coefficient. The values of *n*_CH_ exceeding 100 % can be due to additional ozone decomposition by manganese in its oxide form.

Judging from the results of kinetic investigations of ozone decomposition by $\mathrm{M}\mathrm{n}{\left(\mathrm{L}\right)}_2/\overline{\mathrm{Si}}$ complexes, the nature of the ligands considerably affects the kinetic and stoichiometric parameters of the reaction. Since the nanocompositions obtained as a result of the synthesis differ in their manganese(II) content, it can be concluded that the immobilized Schiff bases differ in their affinity to metal ions. To compare the activity of complexes, the value of *k*_1_ was calculated for 1 mol of the manganese ion. The catalytic activity of isostructural pseudotetrahedral bis-ligand complexes, $\mathrm{M}\mathrm{n}{\left(\mathrm{L}\right)}_2/\overline{\mathrm{Si}}$ (L = L1–L5), determined in such a way, increases in the order Mn(L3)_2_ >> Mn(L4)_2_ > Mn(L1)_2_ > Mn(L2)_2_ > Mn(L5)_2_. The substitution of L4 for L6 changes the geometry of the Mn(II) coordination polyhedron from pseudotetrahedral (Scheme 1) to pseudooctahedral (Scheme 2). This change in the complex structure results in the more smooth decrease in the reaction rate at the beginning of the reaction for $\mathrm{M}\mathrm{n}{\left(\mathrm{L}6\right)}_2/\overline{\mathrm{Si}}$ than for $\mathrm{M}\mathrm{n}{\left(\mathrm{L}4\right)}_2/\overline{\mathrm{Si}}$ (Fig. 2a, b); the *τ*_1/2_ parameter for the first complex is eight times as much as that for the second complex (Table 3), and therefore, the steady-state mode of ozone decomposition is attained sooner for $\mathrm{M}\mathrm{n}{\left(\mathrm{L}4\right)}_2/\overline{\mathrm{Si}}$ (Fig. 2). The mentioned data allow us to conclude that the structure factor has some influence on the kinetics of ozone decomposition and reactivity of the complexes, but its effect is not governing. More likely, a determining factor is the effect of the redox potential of the Mn^3+^/Mn^2+^ pair on the reaction rate constant of ozone decomposition by the $\mathrm{M}\mathrm{n}{\left(\mathrm{L}\right)}_2/\overline{\mathrm{Si}}$ complexes. Judging from the data obtained for crystalline complexes of manganese(II), this redox potential depends on the nature of ligands and substituents in both the aldehyde [26, 28, 36–39] and imine [40] components of the complexes. Because of some difficulties in the process of measuring redox potentials of nanosilica-immobilized manganese complexes, the influence of electronic effects of substituents on the reaction rate constant at the beginning of the reaction was analyzed using the classic Hammett equation. The catalytic activity of $\mathrm{M}\mathrm{n}{\left(\mathrm{L}\right)}_2/\overline{\mathrm{Si}}$ complexes decreases in the order of ligands L4 > L1 > L2 > L5, and that agrees with the increase in electron-acceptor properties of substituents in their benzene rings. In conformity with the Hammett equation, a linear dependence with the negative ρ value (−0.61) has been obtained. Such a dependence demonstrates the increase in the electron density on the central atom and the decrease in the reactivity of the complexes towards a strong oxidizer, namely, ozone.

## Conclusions

The investigations have shown that the oxidation state of manganese(II) does not change upon the synthesis of its complexes with nanosilica-immobilized Schiff bases. L1–L5 ligands form the complexes with the same pseudotetrahedral coordination polyhedron, N_2_O_2_. The L6 ligand, because of its steric factors, forms the pseudooctahedral polyhedron, N_2_Y_4_ (Y—other ligands, namely, H_2_O, Cl^−^), and its bond with the central atom is realized only through nitrogen of imine group.

Nanocomposites represented by the immobilized Schiff bases, $\mathrm{L}/\overline{\mathrm{Si}}$, and manganese(II) complexes, $\mathrm{M}\mathrm{n}{\left(\mathrm{L}\right)}_2/\overline{\mathrm{Si}}$, demonstrated their activity in the reaction of low-temperature ozone decomposition. The reactivity of the nanosilica-immobilized Schiff bases increases in the order L5 < L2 < L3 < L1 < L6 < L4. Manganese(II), as a part of the complexes, shows its catalytic properties in the reaction of ozone decomposition. In the case of the isostructural $\mathrm{M}\mathrm{n}{\left(\mathrm{L}\right)}_2/\overline{\mathrm{Si}}$ complexes (L = L1–L5), the number of catalytic cycles (*n*_Mn_) increases in the order Mn(L3)_2_ >> Mn(L4)_2_ > Mn(L1)_2_ > Mn(L2)_2_ > Mn(L5)_2_. The change in the geometry of a coordination polyhedron in the case of L4 and L6 does not considerably affect the kinetic and stoichiometric parameters of the reaction. The effect of a substituent in the initial aldehyde component of Schiff bases on the reaction rate constant in the case of ozone interaction with the complexes consisting of manganese(II) and L1, L2, L4, and L5 ligands can be described by the Hammett equation with ρ = −0.61.
